# 上海市闵行区低剂量螺旋CT肺癌筛查效果评估

**DOI:** 10.3779/j.issn.1009-3419.2023.102.46

**Published:** 2024-01-20

**Authors:** Jiaoyue TENG, Weiyuan YAO, Weixi LI, Yingling CHENG, Jun LI, Huilin XU, Wanghong XU

**Affiliations:** ^1^200032 上海，复旦大学公共卫生学院（滕姣玥，姚伟元，徐望红）; ^1^Fudan University School of Public Health, Shanghai 200032, China; ^2^201103 上海，上海市闵行区疾病预防控制中心（李为希，程颖玲，李俊，许慧琳）; ^2^Center for Disease Prevention and Control in Minhang District of Shanghai, Shanghai 201103, China

**Keywords:** 肺肿瘤, 低剂量螺旋计算机断层扫描, 筛查, 早期诊断, Lung neoplasms, Low-dose spiral computed tomography, Screening, Early diagnosis

## Abstract

**背景与目的** 低剂量螺旋计算机断层扫描（low-dose spiral computed tomography, LDCT）已被推荐应用于高危人群的肺癌筛查。我国LDCT筛查研究少，随访时间较短，且高危人群的定义不同。本研究基于上海市闵行区肺癌筛查项目，评估LDCT应用于我国人群肺癌筛查的中长期效果。 **方法** 上海市闵行区肺癌筛查项目于2013至2017年实施，共纳入26,124名40岁及以上合格参与者，并以同期闵行区全体适龄人群为对照。通过筛查登记系统及与上海市肿瘤登记和死因登记系统链接，获得所有对象至2020年12月31日的肺癌发病及全死因死亡信息。计算筛查人群相比于全人群对照的肺癌标准化发病率比（standardized incidence ratio, SIR）及95%CI，比较筛查人群与非筛查人群中肺癌病例的早期率（0-I期）、病理类型及5年观察生存率，分析LDCT筛查与肺癌病例全死因死亡的风险比（hazard ratio, HR）及95%CI。 **结果** 筛查人群的肺癌粗发病率为373.3/10万人年（95%CI: 343.1-406.1），标化发病率为70.3/10万人年，相比于总人群的SIR为1.8（95%CI: 1.6-1.9），且在随访期内随时间推移而下降。筛查组病例中早期肺癌占比49.4%，显著高于同期非筛查病例中的38.4%（P<0.05）；肺腺癌占比40.7%，显著高于非筛查病例中的35.9%（P<0.05）；5年观察生存率为53.7%，亦显著高于非筛查病例的41.5%（P<0.05）。与同期非筛查病例相比，筛查组病例的全因死亡风险下降30%（HR=0.7, 95%CI: 0.6-0.8）。 **结论** 本次纳入的筛检人群肺癌发病风险较高；LDCT筛查有利于肺癌的早期检出，提高生存率，在降低人群的肺癌疾病负担方面具有一定潜力。

## Abstract (en)

**Background and objective** Low-dose spiral computed tomography (LDCT) has been recommended for lung cancer screening in high-risk populations. However, evidence from Chinese populations was limited due to the different criteria for high-risk populations and the short-term follow-up period. This study aimed to evaluate the effectiveness in Chinese adults based on the Lung Cancer Screening Program in Minhang District of Shanghai initiated in 2013. **Methods** A total of 26,124 subjects aged 40 years or above were enrolled in the Lung Cancer Screening Program during the period of 2013 and 2017. Results of LDCT examination, and screen-detected cancer cases in all participants were obtained from the Reporting System of the Lung Cancer Screening Program. The newly-diagnosed cases and their vital status up to December 31, 2020 were identified through a record linkage with the Shanghai Cancer Registry and the Shanghai Vital Statistics. Standardized incidence ratio (SIR) and 95%CI were calculated using the local population at ages of 40 or above as the reference. Proportions of early-stage cancer (stage 0-I), pathological types, and 5-year observed survival rates of lung cancer cases were estimated and compared between the cases derived from the screened and non-screened populations. Cox regression models were applied to evaluate the hazard ratio (HR) and 95%CI of LDCT screening with all-cause death of the lung cancer cases. **Results** The crude and age-standardized incidence of lung cancer in screened population were 373.3 (95%CI: 343.1-406.1) and 70.3 per 100,000 person-years, respectively, with an SIR of 1.8 (95%CI: 1.6-1.9), which was observed to decrease with following-up time. The early-stage cancer accounted for 49.4% of all lung cancer cases derived from the screened population, significantly higher than 38.4% in cases from the non-screened population during the same period (P<0.05). The proportion of lung adenocarcinoma (40.7% vs 35.9%) and 5-year survival rate (53.7% vs 41.5%) were also significantly higher in the cases from the screened population (all P<0.05). LDCT screening was associated with 30% (HR=0.7, 95%CI: 0.6-0.8) reduced all-cause deaths of the cases. **Conclusion** The participants of the screening program are at high-risk of lung cancer. LDCT favors the early-detection of lung cancer and improves 5-year survival of the screened cases, indicating a great potential of LDCT in reducing the disease burden of lung cancer in Chinese populations.

肺癌是全球发病率第二、死亡率第一的恶性肿瘤，2020年全球肺癌确诊人数约占新发癌症总人数的11.4%，死亡人数占癌症总死亡人数的18.0%^[1]^。肺癌也是我国最常见的癌症之一，位居癌症死因首位^[2]^。国际早期肺癌行动计划（International Early Lung Cancer Program, I-ELCAP）^[3]^表明，临床I期肺癌经手术治疗后10年生存率可达90%左右，然而，大部分肺癌病例确诊时已处于中晚期。我国2012至2014年III-IV期肺癌占比高达64.6%^[4]^，晚期肺癌患者的5年生存率往往不足10%，整体5年生存率仅19.7%^[5]^，带来严重的疾病负担。急需开展有效的肺癌筛查，提高早期检出率，降低死亡率，改善患者生存状况。

低剂量螺旋计算机断层扫描（low-dose spiral computed tomography, LDCT）可提高肺癌早期检出，改善患者预后及生存期，降低肺癌死亡率^[6⇓⇓-9]^。然而，基于我国人群的LDCT筛查研究不多，大部分报道样本量较小，且采用不同的肺癌高危人群纳入标准，所定义的筛查人群年龄范围在45-80岁不等，吸烟量定义为≥20或≥30包/年，部分研究还纳入了被动吸烟、肺癌家族史等其他危险因素^[8,10⇓⇓⇓-14]^，为评估LDCT的筛查效果带来一定的挑战。

上海是我国较早采用LDCT实施肺癌筛查的试点地区，但迄今仅对筛查项目的初步结果^[10,15,16]^进行了分析和报道，尚缺少对中远期筛查效果的评估。本研究依托上海市闵行区开展的LDCT肺癌筛查项目，开展真实世界研究，随访观察2013至2017年筛查人群中的肺癌发病情况及确诊患者的生存情况，进而对所筛选的高危人群及LDCT的筛查效果进行评估。研究结果将为在我国开展肺癌筛查提供重要的实证依据，并为进一步制定有效的肺癌筛查与早诊早治策略提供参考依据。

## 1 资料与方法

### 1.1 上海市闵行区肺癌筛查项目

上海市闵行区肺癌筛查项目始于2013年，采用机会性筛查方法，为社区居民提供年度LDCT筛查。将肺癌高危对象定义为具备以下任一危险因素：（1）每天吸烟20支以上；（2）有肺癌家族史；（3）有石棉及砷、镍、铬、氡等职业接触史；（4）现患或有肺结核、气道阻塞性疾病史；（5）有咳嗽、咳痰、痰血、胸痛、胸闷等慢性症状^[15,17,18]^。

项目实施期间，由社区卫生中心的公共卫生专业人员动员辖区社区居民自愿参加。所有对象均在知情自愿情况下，于指定医疗机构至少接受1次LDCT筛查，部分对象在研究期间接受2次及以上检查（图1）。LDCT检查采用西门子AS-128型CT机进行，扫描参数为120 kV、30 mA，常规扫描重建层厚5 mm，薄层重建层厚1.5 mm。检查结果由影像科医师根据肺实质和支气管腔内所显示结节的位置、形状、性质和大小进行初步评估，将结节分为实性结节、部分实性结节和非实性结节。既往研究^[14,19]^通常将显示直径4-6 mm及以上结节定义为LDCT检查阳性。本次以至少发现1个直径≥4 mm非钙化结节或肿块作为LDCT筛查阳性^[14,20]^，进一步结合临床表现，将有可疑结节的阳性患者转诊至专科医院进行复诊和确认，并由社区公共卫生专业人员进行随访和追踪观察。项目年度居民纳入情况及随访时间如图2所示。

**图 1:**
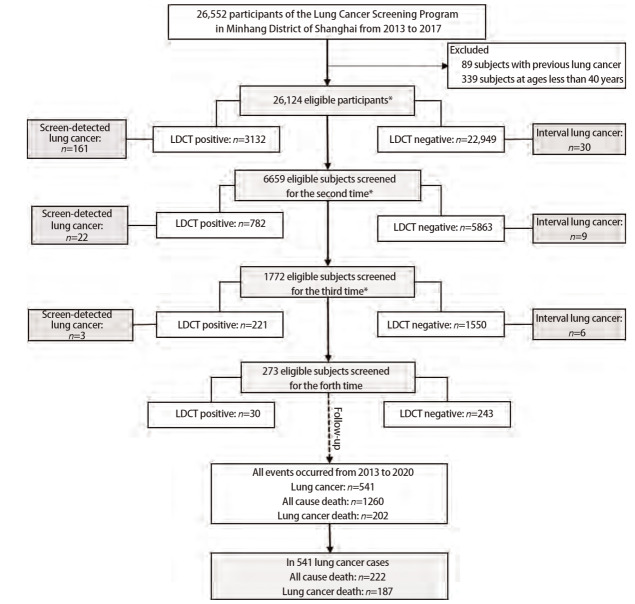
上海市闵行区肺癌筛查项目人员纳入流程图。 *首轮中43人缺失筛查诊断结果，第二轮中14人缺失筛查诊断结果，第三轮中1人缺失筛查诊断结果。

**图 2:**
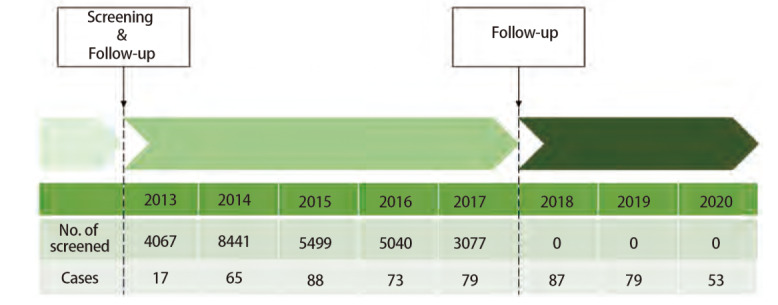
上海市闵行区肺癌筛查项目年度人员纳入及随访情况示意图

### 1.2 研究对象

本研究采用回顾性队列研究设计，以2013至2017年陆续入组参加筛查的26,124名合格人群为对象，建立筛查人群队列。该人群中12,194名是从闵行区社区招募的年龄≥40岁、无肺癌史的居民，13,930名是来自前期所建闵行区肺癌高危库的成员^[10]^。

以研究对象首次参加筛查的日期为起点，随访至2020年12月31日终止。以肺癌确诊、全死因死亡或随访截止3个事件中较早发生者的日期为随访终点，以肺癌筛查及随访期间（2013至2020年）上海市闵行区40岁及以上户籍人群作为全人群对照。

### 1.3 资料来源

研究对象的基线信息由社区医师采用统一的调查问卷进行收集，内容包括一般人口学信息（年龄、性别、受教育程度、收入、职业和婚姻状况）、肺癌危险因素暴露情况（吸烟状况、肺部慢性疾病史、职业暴露史）和肺癌家族史。调查数据、LDCT检查结果及检出肺癌患者信息均来自项目登记系统。

从上海市肿瘤登记系统获得2013至2020年闵行区40岁及以上户籍人口所有新发肺癌病例（ICD-10: C34），并摘录性别、出生年月、加密身份证号、确诊日期、临床分期、病理类型等信息。基于身份证号，将这些肺癌病例与筛查人群进行记录联动，补充筛查人群随访期间的肺癌确诊信息。同样，基于加密身份证号，将筛查人群及所有肺癌病例与上海死因登记系统进行链接，获得全死因死亡及肺癌死亡信息。将2013至2020年筛查人群中发生的肺癌病例定义为“筛查病例”，将去除“筛查病例”后所有闵行区适龄人群中发生的肺癌病例定义为“非筛查病例”。

### 1.4 统计学分析

计数资料以例数和构成比（%）表示，组间差异采用χ²或Fisher's精确法进行比较。粗发病率为肺癌发病人数与观察人年数的比率，用于描述人群的实际发病率水平。标化发病率基于世界标准人口的年龄构成，采用直接法计算，用于去除年龄构成不同产生的影响，比较不同人群的发病风险。筛查人群的肺癌标准化发病率比（standardized incidence ratio, SIR）及95%CI以闵行区同期全人群年龄别肺癌发病率为参照，用于确定筛查人群的发病风险是否与全人群一致。

采用Kaplan-Meier法计算两组患者的5年观察生存率（observed survival rate, OSR）。采用基于逆概率加权（inverse probability weighting, IPW）边际法（marginal）平衡各组年龄与性别分布的差异，绘制校正后的生存率曲线。多因素Cox比例风险模型用于估计LDCT筛查与肺癌病例全死因死亡的风险比（hazard ratio, HR）及95%CI。

所有数据分析采用R 4.2.1统计软件进行，使用双侧检验，以P<0.05为差异有统计学意义。

## 2 结果

### 2.1 筛查对象的基本特征及筛查结果

26,124名筛查对象的中位年龄为64岁[四分位距（interquartile range, IQR）：59-69]，以55-74岁为主，占78.6%；其中男性14,933人，占57.2%。根据肺癌高危人群定义，13,930名参与者被认为是高风险人群，占53.3%。筛查人群中19,447人仅参加了1次筛查，6,659人参加了2次及以上筛查，总筛查人次数为34,828（图1）。多次参加筛查者若后续被确诊为肺癌，则以确诊前最近一次LDCT检查情况作为该对象的筛查结果。

筛查结果如表1所示，26,124名筛查对象共接受LDCT检查34,828次，其中43人次首轮筛查及15人次重复筛查未获取到筛查诊断结果。共发生4165次LDCT阳性（阳性率12.0%），其中186名阳性者在筛查1年内确诊肺癌，检出率为5.3‰，另有45名LDCT阴性者1年内被确诊肺癌，间隔期肺癌占比8.3%。在本次筛查人群中，LDCT首轮筛查的灵敏度为84.3%（95%CI: 79.1%-89.5%），特异度为88.5%（95%CI: 88.1%-88.9%）。

**表 1:** 上海市闵行区26,124名肺癌筛查者的LDCT筛查结果

Index	No. of tests	Positive rate, n (%)	Detection rate, n (‰)	No. of interval cases	Sensitivity (%) (95%CI)	Specificity (%) (95%CI)	Positive predictive value (%)
All tests	34,828	4165 (12.0)	186 (5.3)	45	80.5 (75.4-85.6)	88.5 (88.1-88.8)	4.5
Times of screening							
First time	26,124	3132 (12.0)	161 (6.2)	30	84.3 (79.1-89.5)	88.5 (88.1-88.9)	5.1
Multiple times	8704	1033 (11.9)	25 (2.9)	15	62.5 (47.5-77.5)	88.3 (87.7-89.0)	2.4
Gender							
Male	19,842	2416 (12.2)	99 (5.0)	32	75.6 (68.2-82.9)	88.2 (87.8-88.7)	4.1
Female	14,986	1749 (11.7)	87 (5.8)	13	87.0 (80.4-93.6)	88.8 (88.3-89.3)	5.0
Age (yr)							
40-54	3744	325 (8.7)	9 (2.4)	1	90.0 (71.4-100.0)	91.5 (90.6-92.4)	2.8
55-74	27,715	3336 (12.0)	158 (5.7)	35	81.9 (76.4-87.3)	88.4 (88.1-88.8)	4.7
≥75	3369	504 (15.0)	19 (5.6)	9	67.9 (50.6-85.2)	85.5 (84.3-86.7)	3.8
At high risk of lung cancer							
Yes	19,308	2361 (12.2)	118 (6.1)	27	81.4 (75.0-87.7)	88.3 (87.8-88.7)	5.0
No	15,520	1804 (11.6)	68 (4.4)	18	79.1 (70.5-87.7)	88.7 (88.2-89.2)	3.8

### 2.2 筛查人群随访期间肺癌的发病与死亡情况

对所有筛查对象中位随访5.7年（IQR: 4.6-6.7）后，共确诊肺癌541例，肺癌粗发病率为373.3/10万人年（95%CI: 343.1-406.1），标化发病率为70.3/10万人年（表2）。高危人群发病率较高，粗发病率和标化发病率分别为430.9/10万人年（95%CI: 388.0-478.5）和82.1/10万人年。接受LDCT筛查1年内确诊肺癌最多，占随访期间所有新发病例的35.5%（192/541）。随访期间，筛查人群中共发生1260例全死因死亡，其中202例死于肺癌，肺癌粗死亡率为138.0/10万人年（95%CI: 120.2-158.4），标化死亡率为26.2/10万人年。

**表 2:** 接受LDCT筛查人群的肺癌发病率、标化发病比以及肺癌死亡率

Characteristics	n (%)	Person-year of follow-up	Incidence of lung cancer					Mortality of lung cancer			
			n (%)	Crude (95%CI) (1/100,000)	Age-standardized (1/100,000)	SIR (95%CI)		n (%)		Crude (95%CI) (1/100,000)	Age-standardized (1/100,000)
All participants	26,124	144,924	541	373.3 (343.1-406.1)	70.3	1.8 (1.6-1.9)		202		138.0 (120.2-158.4)	26.2
Gender											
Male	14,933 (57.2)	83,877	371 (68.6)	442.3 (399.5-489.7)	81.3	1.6 (1.4-1.8)		185 (91.6)		218.6 (189.2-252.5)	38.8
Female	11,191 (42.8)	61,047	170 (31.4)	278.5 (239.6-323.6)	54.0	1.8 (1.5-2.1)		17 (8.4)		27.5 (17.1-44.3)	8.0
Age (yr)											
40-54	2836 (10.9)	16,578	16 (3.0)	96.5 (59.1-157.5)	9.4	1.4 (0.7-2.0)		4 (2.0)		24.0 (9.0-64.1)	3.7
55-74	20,541 (78.6)	113,936	443 (81.9)	388.8 (354.2-426.8)	48.4	1.8 (1.7-2.0)		145 (71.8)		125.9 (107.0-148.1)	14.7
≥75	2747 (10.5)	14,410	82 (15.1)	569.0 (458.3-706.6)	12.5	1.6 (1.2-1.9)		53 (26.2)		364.7 (278.6-477.3)	7.8
Educational level*			519					196			
Primary school or below	6512 (26.1)	36,860	172 (33.1)	466.6 (401.9-541.9)	69.8	1.9 (1.6-2.2)		91 (46.4)		244.4 (199.0-300.2)	31.6
High school	17,703 (71.0)	97,701	332 (64.0)	339.8 (305.2-378.4)	69.9	1.7 (1.5-1.9)		101 (51.5)		102.4 (84.3-124.4)	24.9
College or above	716 (2.9)	4002	15 (2.9)	374.8 (225.9-621.7)	66.2	1.5 (0.7-2.3)		4 (2.1)		98.7 (37.0-262.9)	18.7
Monthly income per capital (CNY)*			513					191			
<2000	11,494 (46.2)	64,676	277 (54.0)	428.3 (380.7-481.8)	75.5	1.9 (1.7-2.1)		116 (60.7)		177.5 (148.0-213.0)	29.8
2000-3999	12,019 (48.3)	66,089	211 (41.1)	319.3 (279.0-365.4)	63.3	1.6 (1.3-1.8)		69 (36.1)		103.5 (81.7-131.0)	23.7
≥4000	1356 (5.5)	7466	25 (4.9)	334.8 (226.2-495.5)	68.1	1.8 (1.1-2.6)		6 (3.2)		79.4 (35.7-176.7)	35.2
Marital status*			518					196			
Married	24,113 (96.9)	134,135	497 (95.9)	370.5 (339.3-404.6)	71.9	1.8 (1.6-1.9)		190 (96.9)		140.3 (121.7-161.7)	27.8
Single/Divorced/Widow/Others	775 (3.1)	4206	21 (4.1)	499.3 (325.6-765.8)	75.4	1.9 (1.1-2.7)		6 (3.1)		140.7 (63.2-313.1)	10.8
Occupation*			374					146			
Institution/Government agency	2044 (11.8)	11,605	29 (7.8)	249.9 (173.7-359.6)	64.2	1.3 (0.8-1.8)		14 (9.6)		119.8 (71.0-202.3)	35.7
State-owned enterprise	5018 (29.0)	27,634	117 (31.3)	423.4 (353.2-507.5)	81.4	1.9 (1.6-2.3)		36 (24.7)		128.9 (93.0-178.7)	20.6
Private enterprise	1125 (6.6)	6291	21 (5.6)	333.8 (217.7-512.0)	89.0	1.8 (1.0-2.5)		10 (6.8)		157.7 (84.8-293.1)	57.7
Self-employed/Farmer/Others	5984 (34.6)	33,914	138 (36.9)	406.9 (344.4-480.8)	70.1	1.8 (1.5-2.1)		59 (40.4)		172.3 (133.5-222.4)	23.3
Unemployed	3108 (18.0)	17,338	69 (18.4)	398.0 (314.3-503.9)	61.1	1.7 (1.3-2.0)		27 (18.5)		154.0 (105.6-224.6)	15.6
Smoking*			526					197			
Ever	5283 (21.0)	30,920	125 (23.8)	404.3 (339.3-481.7)	80.3	2.1 (1.7-2.5)		58 (29.4)		186.1 (143.8-240.7)	40.6
Never	19,912(79.0)	109,077	401 (76.2)	367.6 (333.4-405.4)	68.8	1.7 (1.5-1.8)		139 (70.6)		126.1 (106.8-148.9)	22.8
Heavy smoker*^#^			526					197			
Yes	2562 (10.2)	15,173	46 (8.7)	303.2 (227.1-404.8)	71.3	1.8 (1.2-2.3)		19 (9.6)		124.4 (79.3-195.0)	41.7
No	22,633 (89.8)	124,824	480 (91.3)	384.5 (351.6-420.5)	71.0	1.8 (1.6-1.9)		178 (90.4)		141.2 (121.9-163.5)	24.7
Previous COPD or tuberculosis*			526					197			
Ever	1255 (5.0)	7429	27 (5.1)	363.4 (249.2-529.9)	54.9	1.5 (0.9-2.1)		12 (6.1)		160.1 (90.9-282.0)	19.4
Never	23,917 (95.0)	132,434	499 (94.9)	376.8 (345.1-411.3)	72.5	1.8 (1.6-1.9)		185 (93.9)		138.3 (119.8, 159.8)	27.5
Family history of lung cancer*			526					197			
Yes	2,053 (8.1)	12,245	33 (6.3)	269.5 (191.6-379.1)	52.8	1.5 (1.0-2.0)		6 (3.0)		48.5 (21.8-107.9)	9.2
No	23,142 (91.9)	127,751	493 (93.7)	385.9 (353.3-421.5)	72.3	1.8 (1.6-1.9)		191 (97.0)		148.1 (128.5-170.6)	28.3
Occupational exposures*			518					194			
Ever	151 (0.6)	868	2 (0.4)	230.4 (57.6-921.3)	31.4	-		1 (0.5)		114.6 (16.1-813.3)	15.5
Never	24,834 (99.4)	137,953	516 (99.6)	374.0 (343.1-407.7)	71.0	1.8 (1.6-1.9)		193 (99.5)		138.5 (120.3-159.5)	26.6
At high risk of lung cancer											
Yes	13,930 (53.3)	81,229	350 (64.7)	430.9 (388.0-478.5)	82.1	2.0 (1.8-2.2)		149 (73.8)		181.5 (154.5-213.1)	34.3
No	12,194 (46.7)	63,695	191 (35.3)	299.9 (260.2-345.6)	55.3	1.4 (1.2-1.6)		53 (26.2)		82.5 (63.0-108.0)	15.8
Years of follow-up^&^											
<1	26,124	25,947	192 (35.5)	740.0 (642.4-852.4)	134.7	3.5 (3.0-3.9)					
1-3	25,826	51,258	148 (27.4)	288.7 (245.8-339.2)	55.2	1.4 (1.1-1.6)					
3-5	25,404	44,023	128 (23.7)	290.8 (244.5-345.8)	58.0	1.4 (1.1-1.6)					
>5	17,048	23,696	73 (13.5)	308.1 (244.9-387.5)	54.6	1.5 (1.2-1.8)					

*Missing values of 1193 for educational level, 1255 for income, 1236 for marital status, 8845 for occupation, 929 for smoking and family history of lung cancer, 952 for previous COPD or tuberculosis, and 1139 for occupational exposure. ^#^Heavy smoker: Current or former smokers who have ever had at least 20 pack-years of cigarette smoking. ^&^Years of follow-up: Number of participants in the screening cohort during different follow-up periods. COPD: chronic obstructive pulmonary disease.

同期闵行区共确诊40岁及以上肺癌病例8838例，粗发病率和标化率分别为173.8/10万人年和43.2/10万人年。与全人群相比，筛查人群总体SIR为1.8（95%CI: 1.6-1.9），其中男性为1.6（95%CI: 1.4-1.8），女性为1.8（95%CI: 1.5-2.1）。筛查人群的肺癌发病风险高于同期当地人群，筛查后的第1年肺癌SIR高达3.5（95%CI: 3.0-3.9），而后有较大幅度的下降，随后略上升。

### 2.3 “筛查病例”与“非筛查病例”的临床分期及病理类型比较

将0-I期肺癌定义为早期，并按组织学分类，将肺癌分为小细胞肺癌（small cell lung cancer, SCLC）和非小细胞肺癌（non-small cell lung cancer, NSCLC），后者主要包括肺鳞状细胞癌（lung squamous cell carcinoma, LUSC）和肺腺癌（lung adenocarcinoma, LUAD）。如表3所示，筛查人群中有121人被确诊为早期肺癌，早期率为49.4%，显著高于同期“非筛查病例”38.4%的早期率（χ²=38.7, P<0.05），且男、女性“筛查病例”均具有更高的早期率。“筛查病例”与“非筛查病例”的病理类型亦有显著差异（χ²=9.2, P<0.05）。“筛查病例”中LUSC的占比为5.5%，略低于“非筛查病例”中的6.2%，而“筛查病例”中LUAD占比40.7%，显著高于“非筛查病例”中的35.9%（P<0.05），差异在女性中更为明显（64.1% vs 48.2%, P<0.05）。

**表 3:** 上海市闵行区筛查组肺癌病例与对照人群中肺癌病例的分期和组织学类型比较

Histologic type	Cases from screened population							Cases from remaining residents					
	n (%)	Stage of lung cancer [n (%)]						n (%)	Stage of lung cancer [n (%)]				
		0-I	II	III	IV	Unknown			0-I	II	III	IV	Unknown
All subjects, n (%)	541	121 (49.4)	16 (6.5)	30 (12.2)	78 (31.9)	296		8373	1124 (38.4)	219 (7.5)	387 (13.2)	1195 (40.9)	5448
SCLC	25 (4.6)	2	0	2	6	15		273 (3.3)	7	9	36	79	142
LUAD	220 (40.7)	90	7	11	26	86		3004 (35.9)	890	104	149	549	1312
LUSC	30 (5.5)	3	4	6	6	11		516 (6.2)	52	48	106	110	200
Other NSCLC	266 (49.2)	26	5	11	40	184		4,580 (54.7)	175	58	96	457	3794
Male, n (%)	371	57 (35.4)	12 (7.5)	25 (15.5)	67 (41.6)	210		4999	495 (28.0)	153 (8.7)	308 (17.5)	809 (45.8)	3234
SCLC	22 (5.9)	2	0	1	6	13		252 (5.0)	7	9	33	74	129
LUAD	111 (29.9)	37	3	10	18	43		1377 (27.6)	350	59	101	301	566
LUSC	30 (8.1)	3	4	6	6	11		480 (9.6)	50	45	97	100	188
Other NSCLC	208 (56.1)	15	5	8	37	143		2890 (57.8)	88	40	77	334	2351
Female, n (%)	170	64 (76.2)	4 (4.8)	5 (5.9)	11 (13.1)	86		3374	629 (54.2)	66 (5.7)	79 (6.8)	386 (33.3)	2214
SCLC	3 (1.8)	0	0	1	0	2		21 (0.6)	0	0	3	5	13
LUAD	109 (64.1)	53	4	1	8	43		1627 (48.2)	540	45	48	248	746
LUSC	0 (0.0)	0	0	0	0	0		36 (1.1)	2	3	9	10	12
Other NSCLC	58 (34.1)	11	0	3	3	41		1690 (50.1)	87	18	19	123	1443

SCLC: small cell lung cancer; LUAD: lung adenocarcinoma; LUSC: lung squamous cell carcinoma; NSCLC: non-small cell lung cancer. The distributions of both stage and histologic types show significantly differences between groups (All P<0.05).

### 2.4 “筛查病例”与“非筛查病例”的生存情况

541名“筛查病例”中共发生222例（202例男性，20例女性）全因死亡，其中187例死于肺癌，8373名“非筛查病例”中发生了4008例（2908例男性，1100例女性）全因死亡，其中2706例死于肺癌。图3显示了校正年龄和性别后两组病例的生存曲线。“筛查病例”的5年OSR为53.7%，显著高于“非筛查病例”的41.5%，差异在女性（86.5% vs 57.9%）和男性病例中（37.7% vs 30.7%）均达显著水平。与“非筛查病例”相比，“筛查病例”的全因死亡风险降低了30%（HR=0.7, 95%CI: 0.6-0.8），男性和女性病例分别降低了20%（HR=0.8, 95%CI: 0.7-0.9）和70%（HR=0.3, 95%CI: 0.2-0.5）。

**图 3:**
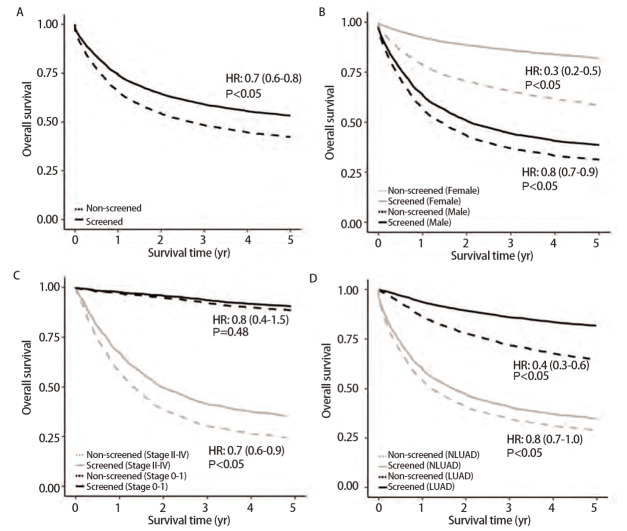
上海市闵行区肺癌筛查和非筛查病例的生存曲线及死亡风险比较。 A：全体病例（调整年龄与性别）；B： 按性别分层（调整年龄）；C：按确诊时肺癌分期分层（调整年龄与性别）；D： 据肺癌的组织学类型分层（调整年龄与性别）。

按肺癌分期进行分层分析发现，“筛查病例”早期肺癌（0-I期）和中晚期肺癌（II-IV期）的5年OSR分别为90.4%和36.7%，分别高于“非筛查病例”中的89.1%和24.5%。相较于“非筛查病例”，早期“筛查病例”全因死亡的HR为0.8（95%CI: 0.4-1.5），两组差异无统计学意义，但在中晚期肺癌病例中观察到“筛查病例”较低的死亡风险（HR=0.7, 95%CI: 0.6-0.9）。

同样，按肺癌病理类型进行分层分析，发现“筛查病例”中LUAD和非腺癌（non-LUAD, NLUAD）的5年OSR均高于“非筛查病例”（LUAD: 82.1% vs 66.0%; NLUAD: 30.2% vs 26.3%），且“筛查病例”的死亡风险显著低于“非筛查病例”（LUAD: HR=0.4, 95%CI: 0.3-0.6; NLUAD: HR=0.8, 95%CI: 0.7-1.0）。

## 3 讨论

本研究以2013至2017年上海市闵行区肺癌筛查项目参加者为对象，以当地同期适龄人群为全人群对照，分析了筛查对象的肺癌发病率、死亡率以及SIR；通过比较筛查人群与非筛查人群中肺癌病例的早期率、病理分型和生存率，评估LDCT的筛查效果。研究结果显示，由于纳入的筛查人群多为肺癌高危者，因此筛查人群有较高的肺癌发病率；进一步分析发现，筛查人群的肺癌发病率随时间推移而下降，提示筛查的时期效应；“筛查病例”的早期率及5年OSR均高于“非筛查病例”，而全死因死亡风险低于“非筛查病例”，这些差别在女性中更明显，证明了LDCT的筛查效果；然而，“筛查病例”中女性早期LUAD占比显著高于“非筛查病例”，提示可能存在肺癌的过度诊断。

本次以LDCT筛查1年内确诊的肺癌作为应检出的病例，将其中LDCT阳性者定义为筛检肺癌，LDCT阴性者定义为间隔期肺癌，所估计的LDCT灵敏度仅为84.3%，远低于美国国家肺癌筛查试验（National Lung Screening Trial, NLST）及荷兰-比利时肺癌筛查试验（Nederlands-Leuvens Longkanker Screenings Onderzoek, NELSON）中同样以1年为应检出时间而得到的93.8%^[21]^和94.6%^[22]^。本次LDCT筛查所采用的扫描仪参数设置为120 kV、30 mA，常规扫描重建层厚5 mm，薄层重建层厚1.5 mm，其诊断性能不同于NLST及NELSON研究所采用的120-140 kV、40-80 mA、1.0-3.2 mm常规扫描重建层厚以及0.7-2.5 mm重建间隔层厚的参数^[23]^。可见LDCT的准确性受所使用设备性能的影响，也可能因影像学医师临床诊断经验和LDCT阳性结果判定标准的不同而异^[24]^，也不排除不同种族和人群之间的差异^[25,26]^。

LDCT筛查阳性率与筛查人群的发病风险相关。本次LDCT的总体阳性率为12.0%，低于在上海市和四川省成都市高危人群中分别报道的22.9%^[14]^和20.4%^[11]^，这与本次筛查项目纳入了部分非高危人群有关。LDCT筛查的阳性率还因判断标准不同而异。本次筛查将检测到直径≥4 mm的非钙化结节或肿块定义为LDCT阳性，所得阳性率高于同样纳入了高危和非高危人群但将检出直径≥5 mm的实性结节或部分实性结节或≥8 mm非实性结节或发现疑似肺癌的气道病变、结节、肿块判定为LDCT阳性的云南省一项研究中9.7%的阳性率^[27]^和深圳人群中4.9%的阳性率^[28]^。

本次肺癌总体检出率为5.3‰，高危人群中的检出率为6.1‰，高于新疆乌鲁木齐采用我国城市癌症早诊早治项目常见癌症风险评估系统所得高危人群中1.7‰的肺癌检出率^[29]^和广东省揭阳市基于我国LDCT肺癌筛查专家共识所得高危人群中5.4‰的检出率^[30]^，接近成都市采用美国国立综合癌症网络（National Comprehensive Cancer Network, NCCN）标准所选肺癌高危人群中6.9‰的基线检出率^[11]^，提示本次制定的高危人群标准较为合理。然而，该检出率低于NLST的10.3‰^[21]^和NLESON试验的8.7‰^[22]^，这可能是因为本次筛查对象中42.8%为女性，重度吸烟者仅占10.2%，不同于欧美国家基于年龄和吸烟量确定的肺癌高风险人群^[6,9,31,32]^。我国吸烟者发生肺癌的风险远低于西方人群，相反，40%以上的肺癌发生在非吸烟人群中^[33]^。肺癌家族史、空气污染、呼吸道疾病和遗传易感性等因素都可能导致肺癌的发病风险升高^[34]^。本次肺癌筛查纳入高危人群时同时考虑了吸烟、肺癌家族史、职业暴露及肺部慢性疾病史，使吸烟率极低但可能暴露于室外空气污染或家庭固体燃料燃烧生成的可吸入物质等危险因素的广大女性得以纳入筛查^[35]^，人群覆盖面更广。多项研究^[36⇓-38]^发现，亚洲女性肺癌发病风险较高，不应该排除在LDCT筛查对象之外。一项荟萃分析^[39]^也观察到LDCT筛查可显著降低非吸烟亚洲女性的肺癌死亡率和全因死亡率。可见，LDCT这一筛查方法适用于我国人群，但有待进一步探索如何更准确选择高危人群，尤其需要识别非吸烟女性肺癌的风险因素。

本次LDCT筛查肺癌的效果主要体现在“筛查病例”的分期较早，早期肺癌的比例较高。值得注意的是，男、女性“筛查病例”中LUAD占比分别达29.9%和64.1%，早期肺癌中LUAD比例更高，提示LDCT可能对LUAD更灵敏。该筛查结果与既往研究^[14,37,40,41]^一致。2012至2018年，上海、江苏、山东和广东6家医院开展的LDCT筛查检出的0-I期早期肺癌占比高达95.0%，其中98.9%为LUAD^[38]^。上海、江苏徐州和澳门特别行政区开展的研究^[14,41,42]^也均发现LDCT筛查有助于识别早期肺癌，对LUAD尤为敏感。

本次LDCT筛查肺癌的效果还体现在“筛查病例”较高的5年生存率。肺癌的生存率随确诊时临床分期的升高而降低，且疾病本身具有高度的异质性，不同病理类型患者的生存率亦有显著差异^[42,43]^。LUAD占NSCLC病例总数的50%-60%，确诊时恶性程度相对较低^[44,45]^。本次女性病例的5年生存率显著高于男性，这与女性中检出较多早期LUAD有关。既往研究表明，亚洲女性可能因检出较多早期LUAD而存在过度诊断^[46,47]^，即尽管筛查发现了早期肺癌，但这部分患者不接受筛查或不经确诊也不会影响寿命^[48]^。本次筛查人群中较多的女性早期LUAD或提示了过度诊断的可能性。然而，本研究是一项回顾性队列研究，无法对筛查所致的过度诊断进行准确评估和量化，有待采用随机对照试验设计，对研究对象进行长期随访，对可能存在的过度诊断进行深入探讨。近年来，循环肿瘤DNA（circulating tumor DNA, ctDNA）和微小残留病灶（minimal residual disease, MRD）等生物标志物越来越多地应用于肺癌预后及复发的预测^[49⇓⇓⇓-53]^，也可能在过度诊断的识别中起一定作用。

本研究采用回顾性队列研究方法，基于上海市闵行区肺癌筛查项目，评估了LDCT在我国肺癌高危与部分非高危人群中的筛查效果。本研究存在一定的局限性。首先，参与筛查者的危险因素信息为自报，可能存在因回忆偏倚和报告偏倚而导致的错分。其次，筛查组肺癌患者较高的5年生存率可部分归因于领先时间偏倚。此外，本研究中筛查组入组截止时间为2017年9月20日，此后小规模人群筛查仍在持续进行中，因此“非筛查病例”中存在部分因筛检而早发现的肺癌病例，可能导致对LDCT筛查效果的低估。

综上所述，本研究结果证实了LDCT筛查在我国重度吸烟率较低、女性占比高的肺癌“高危”人群中有利于肺癌病例的早发现及预后的改善，尚待进一步探讨如何更准确地识别我国人群中的肺癌高危个体，从而更有效地实施LDCT筛查。
